# High quality draft genome sequence of *Bacteroides barnesiae* type strain BL2^T^ (DSM 18169^T^) from chicken caecum

**DOI:** 10.1186/s40793-015-0045-6

**Published:** 2015-08-02

**Authors:** Mitsuo Sakamoto, Alla L. Lapidus, James Han, Stephan Trong, Matthew Haynes, T. B. K. Reddy, Natalia Mikhailova, Marcel Huntemann, Amrita Pati, Natalia N. Ivanova, Rüdiger Pukall, Victor M. Markowitz, Tanja Woyke, Hans-Peter Klenk, Nikos C. Kyrpides, Moriya Ohkuma

**Affiliations:** Microbe Division/Japan Collection of Microorganisms, RIKEN BioResource Center, Tsukuba, Ibaraki Japan; Theodosius Dobzhansky Center for Genome Bionformatics, St. Petersburg State University, St. Petersburg, Russia; Algorithmic Biology Lab, St. Petersburg Academic University, St. Petersburg, Russia; DOE Joint Genome Institute, Walnut Creek, CA USA; Leibniz-Institute DSMZ - German Collection of Microorganisms and Cell Cultures, Braunschweig, Germany; Biological Data Management and Technology Center, Lawrence Berkeley National Laboratory, Berkeley, CA USA; Department of Biological Sciences, Faculty of Science, King Abdulaziz University, Jeddah, Saudi Arabia

**Keywords:** Strictly anaerobic, Non-motile, Rod-shaped, Gram-negative, Cecum, Poultry, *Bacteroidaceae*

## Abstract

*Bacteroides barnesiae* Lan *et al.* 2006 is a species of the genus *Bacteroides*, which belongs to the family *Bacteroidaceae*. Strain BL2^T^ is of interest because it was isolated from the gut of a chicken and the growing awareness that the anaerobic microbiota of the caecum is of benefit for the host and may impact poultry farming. The 3,621,509 bp long genome with its 3,059 protein-coding and 97 RNA genes is a part of the Genomic Encyclopedia of Type Strains, Phase I: the one thousand microbial genomes (KMG) project.

## Introduction

Strain BL2^T^ (= DSM 18169 = CCUG 54636 = JCM 13652) is the type strain of *Bacteroides barnesiae* which belongs to the genus *Bacteroides* [1]. The species epithet is derived from the name of Ella M. Barnes, a British microbiologist, who has contributed much to our knowledge of intestinal bacteriology and anaerobic bacteriology in general. *B. barnesiae* strain BL2^T^ was isolated from caecum of a healthy chicken. Four other strains belonging to the same species have been isolated from the same source [1]. The genus *Bacteroides* represents one of the predominant anaerobic genera found in chicken caecum [2–4]. *Bacteroides* species are thought to play a fundamental role in the breakdown of complex molecules (such as polysaccharides) into simpler compounds that are used by the animal host as well as the microorganisms themselves [5, 6], in the utilization of nitrogenous substances and in the biotransformation of bile acids and other steroids [7]. They also play a role as beneficent protectors of the gut against pathogenic microorganisms [8]. Here we present a summary classification and set of features for *B. barnesiae* strain BL2^T^, together with the description of the complete genomic sequencing and annotation.

## Organism information

### Classification and features

**Fig. 1:**
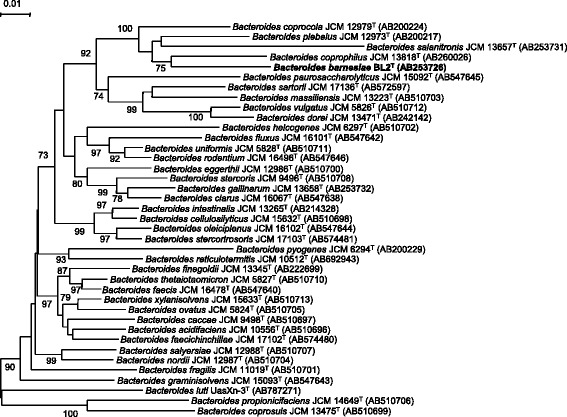
Phylogenetic tree based on the 16S rRNA gene sequences showing the relationship of *Bacteroides barnesiae* strain BL2^T^ among the genus *Bacteroides* . The tree was constructed by the neighbor-joining method. Numbers at nodes indicate the percentage bootstrap values of 1000 replicates. Bars, 0.01 substitutions per nucleotide position. Accession numbers are given for each strain

A 1301 bp long contig contained the most complete 16S rRNA gene copy in the draft genome. This partial gene differed by 7 nucleotides (0.5 %) from the 16S rRNA reference sequence (AB253726) generated for the original description of *B. barnesiae* [1]. Such a difference is not unusual when comparing original sequences from the time organisms were initially described with sequences of type strain genomes sequenced in the KMG project [9], a problem that was only partially resolved in the sequencing orphan species initiative (SOS) [10]. A representative 16S rRNA gene sequence of strain BL2^T^ was compared with GenBank using NCBI BLAST. The single most frequent genus found was *Bacteroides*. The highest-scoring environmental sequences (up to 99.8 % sequence identity), including HQ784912 (‘gastrointestinal specimens clone ELU0102-T240-S-NI_000093’), were all from a study on gastrointestinal specimens linked to inflammatory bowel diseases phenotype in human ileum [11] and indicate that close relatives of strain BL2^T^ and representatives of *B. barnesiae* are also relevant to human health. Fig. 1 shows the phylogenetic position of *B. barnesiae* in a 16S rRNA gene sequence-based tree.

**Fig. 2:**
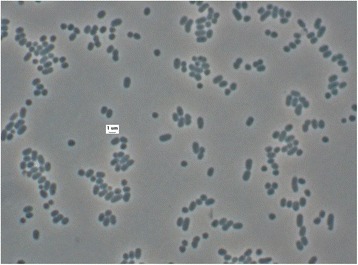
Light microscope image of strain BL2^T^

The cells of *B. barnesiae* are pleomorphic rods (0.5-1.4 × 0.8-10.6 μm) (Fig. 2). The cells are usually arranged singly or in pairs [1]. *B. barnesiae* is a Gram-negative, non-sporeforming bacterium (Table 1) that is described as non-motile, with only seven genes associated with motility having been found in the genome (see below). The optimum temperature for growth of strain BL2^T^ is 37 °C. *B. barnesiae* is a strictly anaerobic chemoorganotroph and is able to ferment glucose, lactose, sucrose, maltose, salicin, xylose, cellobiose, mannose and raffinose [1]. The organism hydrolyzes esculin but does not liquefy gelatin, and neither reduces nitrate nor produces indole from tryptophan [1]. *B. barnesiae* does not utilize mannitol, arabinose, glycerol, melezitose, sorbitol, rhamnose or trehalose [1]. Growth is possible in the presence of bile [1]. Major fermentation products from broth (1 % peptone, 1 % yeast extract, and 1 % glucose each (w/v)) are acetic acid and succinic acid, whereas isovaleric acid is produced in small amounts [1]. *B. barnesiae* shows activity for α-galactosidase, β-galactosidase, α-glucosidase, β-glucosidase, α-arabinosidase, *N*-acetyl-β-glucosaminidase, α-fucosidase, alkaline phosphatase, leucyl glycine arylamidase, alanine arylamidase and glutamyl glutamic acid arylamidase but no activity urease, catalase, arginine dihydrolase, β-galactosidase 6-phosphate, β-glucuronidase, glutamic acid decarboxylase and arginine, proline, phenylalanine, leucine, pyroglutamic acid, tyrosine, glycine, histidine and serine arylamidase [1].

**Table 1:** Classification and general features of *Bacteroides barnesiae* strain BL2^T^ in accordance with the MIGS recommendations [33] published by the Genome Standards Consortium [34] and the NamesforLife database [35]

MIGS ID	Property	Term	Evidence code
	Current classification	Domain *Bacteria*	TAS [36]
		Phylum *Bacteroidetes*	TAS [37, 38]
		Class *Bacteroidia*	TAS [38, 39]
		Order *Bacteroidales*	TAS [38, 40]
		Family *Bacteroidaceae*	TAS [41, 42]
		Genus *Bacteroides*	TAS [42, 43]
		Species *Bacteroides barnesiae*	TAS [1]
		Strain BL2^T^	TAS [1]
	Gram stain	Negative	TAS [1]
	Cell shape	Pleomorphic rods	TAS [1]
	Motility	Non-motile	TAS [1]
	Sporulation	Non-sporulating	TAS [1]
	Temperature range	Mesophilic	TAS [1]
	Optimum temperature	37 °C	TAS [1]
	pH range; Optimum	Not reported	
	Carbon source	Mono- and polysaccharides	TAS [1]
	Energy metabolism	Chemoorganotroph	TAS [1]
MIGS-6	Habitat	Chicken	TAS [1]
MIGS-6.3	Salinity	Not reported	
MIGS-22	Oxygen requirement	Strictly anaerobic	TAS [1]
MIGS-15	Biotic relationship	Free-living	TAS [1]
MIGS-14	Pathogenicity	None	NAS
	Biosafety level	1	NAS
MIGS-23	Isolation	Chicken caecum	TAS [1]
MIGS-4	Geographic location	Japan	TAS [1]
MIGS-5	Sample collection time	Not reported	
MIGS-4.1	Latitude	Not reported	
MIGS-4.2	Longitude	Not reported	
MIGS-4.3	Depth	Not reported	
MIGS-4.4	Altitude	Not reported	

Evidence codes - TAS: Traceable Author Statement (i.e., a direct report exists in the literature); NAS: Non-traceable Author Statement (i.e., not directly observed for the living, isolated sample, but based on a generally accepted property for the species, or anecdotal evidence). These evidence codes are from the Gene Ontology project [44]

*B. barnesiae* strain BL2^T^ contains menaquinones MK-10 (58 %) and MK-11 (34 %) as principal respiratory quinones, small amounts of MK-8, MK-9 and MK-12 (2 % each) are found as minor components [1]. The major fatty acids found were *anteiso*-C_15:0_ (32 %), *iso*-C_15:0_ (15 %), 3-hydroxy C_16:0_ (10 %) and C_16:0_ (10 %). Fatty acids C_14:0_ (4 %), C_15:0_ (2 %), C_18:1_*ω*9*c* (4 %), C_18:2_*ω*6,9*c* (2 %) and 3-hydroxy *iso*-C1_7:0_ (7 %) were found in minor amounts [1]. Chemotaxonomic features are in line with known features from other representatives of the genus [1].

## Genome sequencing information

### Genome project history

The organism was selected for sequencing on the basis of its phylogenetic position [12–14]. Sequencing of *B. barnesiae* strain BL2^T^ is part of Genomic Encyclopedia of Type Strains, Phase I: the one thousand microbial genomes project [9] which aims not only to increase the sequencing coverage of key reference microbial genomes [15], but also to generate a large genomic basis for the discovery of genes encoding novel enzymes [16]. The genome project is deposited in the Genomes OnLine Database [17] and the permanent draft genome sequence is deposited in GenBank. Sequencing, finishing and annotation were performed by the DOE Joint Genome Institute using state of the art sequencing technology [18]. A summary of the project information is shown in Table 2.

**Table 2:** Genome sequencing project information

MIGS ID	Property	Term
MIGS-31	Finishing quality	Level 2: High-Quality Draft
MIGS-28	Libraries used	Illumina Std. shotgun library
MIGS-29	Sequencing platforms	Illumina HiSeq 2000
MIGS-31.2	Fold coverage	122.7 ×
MIGS-30	Assemblers	Velvet v. 1.1.04; ALLPATHS v. r41043
MIGS-32	Gene calling method	Prodigal
	Locus Tag	C510
	Genbank ID	ARGC00000000
	Genbank Date of Release	16-SEP-2013
	GOLD ID	Gi11191
	BIOPROJECT	PRJN174979
MIGS-13	Source Material Identifier	DSM 18169
	Project relevance	Tree of Life, GEBA-KMG

### Growth conditions and genomic DNA preparation

*B. barnesiae* strain BL2^T^, DSM 18169, was grown anaerobically in DSMZ medium 429 (Columbia Blood Agar) at 37 °C [19]. DNA was isolated from 0.5-1 g of cell paste using JetFlex genomic DNA purification (GENOMED) following the standard protocol as recommended by the manufacturer with and additional protease K (50 μl; 21 mg/ml) digest for 60 min. at 58 °C followed by addition of 200 μl Protein Precipitation Buffer after protein precipitation and overnight incubation on ice. DNA is available through the DNA Bank Network [20].

### Genome sequencing and assembly

The permanent draft genome of *B. barnesiae* strain BL2^T^ was generated using Illumina technology [18, 21]. An Illumina Standard shotgun library was constructed and sequenced using the Illumina HiSeq 2000 platform which generated 11,109,700 reads totaling 1,666.5 Mb. All general aspects of library construction and sequencing performed at the DOE-JGI can be found at [22]. All raw Illumina sequence data was passed through DUK, a filtering program developed at JGI, which removes known Illumina sequencing and library preparation artifacts [23]. Following steps were then performed for assembly: (1) filtered Illumina reads were assembled using Velvet [24], (2) 1–3 kb simulated paired end reads were created from Velvet Contigs using wgsim [25], (3) Illumina reads were assembled with simulated read pairs using Allpaths–LG (version r41043) [26]. Parameters for assembly steps were: 1) Velvet (velveth: 63 –shortPaired and velvetg: −very clean yes –export- Filtered yes –min contig lgth 500 –scaffolding no –cov cutoff 10) 2) wgsim (−e 0 –1 100 –2 100 –r 0 –R 0 –X 0) 3) Allpaths–LG (PrepareAllpathsInputs: PHRED 64 = 1 PLOIDY = 1 FRAG COVERAGE = 125 JUMP COVERAGE = 25 LONG JUMP COV = 50, RunAllpathsLG: THREADS = 8 RUN = std shredpairs TARGETS = standard VAPI WARN ONLY = True OVERWRITE = True). The final draft assembly contained 47 contigs in 43 scaffolds. The total size of the genome is 3.6 Mb and the final assembly is based on 443.6 Mb of Illumina data, which provides an average 122.7 × coverage of the genome.

### Genome annotation

Genes were identified using Prodigal [27] as part of the DOE-JGI genome annotation pipeline [28, 29], following by a round of manual curation using the JGI GenePRIMP pipeline [30]. The predicted CDSs were translated and used to search the National Center for Biotechnology Information non-redundant database, UniProt, TIGR-Fam, Pfam, PRIAM, KEGG, COG, and InterPro database. These data sources were combined to assert a product description for each predicted protein. Additional gene prediction analysis and functional annotation was performed within the Integrated Microbial Genomes-Expert Review platform [31].

## Genome properties

The assembly of the draft genome sequence consists of 43 scaffolds amounting to 3,621,509 bp, and the G + C content is 46.8 % (Table 3). Of the 3,156 genes predicted, 3,059 were protein-coding genes, and 97 RNAs. The majority of the protein-coding genes (71.7 %) were assigned a putative function while the remaining ones were annotated as hypothetical proteins. The distribution of genes into COGs functional categories is presented in Table 4.

**Table 3:** Genome statistics

Attribute	Value	% of total
Genome size (bp)	3,621,509	100.00
DNA coding region (bp)	3,241,163	89.50
DNA G + C content (bp)	1,696,150	46.84
DNA scaffolds	43	
Total genes	3,156	100.00
Protein coding genes	3,059	96.93
RNA genes	97	3.07
Genes with function prediction	2,263	71.70
Genes assigned to COGs	1,668	52.85
Genes with Pfam domains	2,431	77.03
Genes with signal peptides	445	14.10
Genes with transmembrane helices	711	22.53
CRISPR repeats	7

**Table 4:** Number of genes associated with the general COG functional categories

Code	Value	% age	Description
J	144	8.03	Translation, ribosomal structure and biogenesis
A	0	0.00	RNA processing and modification
K	107	5.96	Transcription
L	126	7.02	Replication, recombination and repair
B	0	0.00	Chromatin structure and dynamics
D	20	1.11	Cell cycle control, cell division, chromosome partitioning
Y	0	0.00	Nuclear structure
V	62	3.46	Defense mechanisms
T	60	3.34	Signal transduction mechanisms
M	142	7.72	Cell wall/membrane/envelope biogenesis
N	4	0.22	Cell motility
Z	0	0.00	Cytoskeleton
W	0	0.00	Extracellular structures
U	47	2.62	Intracellular trafficking, secretion, and vesicular transport
O	60	3.34	Posttranslational modification, protein turnover, chaperones
C	103	5.74	Energy production and conversion
G	140	7.30	Carbohydrate transport and metabolism
E	138	7.69	Amino acid transport and metabolism
F	64	3.57	Nucleotide transport and metabolism
H	90	5.02	Coenzyme transport and metabolism
I	48	2.68	Lipid transport and metabolism
P	97	5.41	Inorganic ion transport and metabolism
Q	19	1.06	Secondary metabolites biosynthesis, transport and catabolism
R	219	12.21	General function prediction only
S	104	5.80	Function unknown
-	1,488	47.15	Not in COGs

## Insights from the genome sequence

*B. barnesiae* strain BL2^T^, *Bacteroides salanitronis* strain BL78^T^ and *Bacteroides gallinarum* strain C35^T^ were isolated from the cecum of the same healthy chicken [1]. The GGDC (Genome-to-Genome Distance Calculator) web server (GGDC 2.0) [32] was used for the estimation of the overall similarity between the three *Bacteroides* genomes. The comparison of *B. barnesiae* with *B. salanitronis* and *B. gallinarum* revealed that 11.1 % and 5.2 %, respectively, of the average of the genome lengths are covered with HSPs (high-scoring segment pairs). The identity within the HSPs was 83.6 % and 84.6 %, respectively, whereas the identity over the whole genome was 9.3 % and 4.4 %, respectively. The comparison of *B. gallinarum* with *B. salanitronis* revealed that 5.4 % of the genome is covered with HSPs, with an identity within in the HSPs of 84.1 % and an identity over the whole genome of 4.6 %. According to these calculations the similarity between *B. barnesiae* and *B. salanitronis* is higher than the similarity between *B. barnesiae* and *B. gallinarum* as well as the similarity between *B. gallinarum* and *B. salanitronis*.

The genome size of *B. barnesiae* (3.6 Mb) is significantly smaller than those of *B. salanitronis* (4.3 Mb) and *B. gallinarum* (4.9 Mb).

## Conclusions

*B. barnesiae* strain BL2^T^ genome consists of a single chromosome of 3.6 Mb predicted to encode 3,156 genes. Strain BL2^T^ has a relatively small genome in comparison to other sequenced *Bacteroides* species isolated from the same chicken (4.3-4.9 Mb). These differences of genome size may be the results of adaptation in this niche. Further study will be necessary for elucidation of this idea.

## Abbreviations

KMG: One thousand microbial genomes
JGI: Joint genome institute
SOS: Sequencing orphan species
GGDC: Genome-to-genome distance calculator
